# HGF/MET-directed therapeutics in gastroesophageal cancer: a review of clinical and biomarker development

**DOI:** 10.18632/oncotarget.2003

**Published:** 2014-05-24

**Authors:** Stephen P. Hack, Jean-Marie Bruey, Hartmut Koeppen

**Affiliations:** ^1^ Product Development, Genentech Inc., South San Francisco, CA, USA; ^2^ Oncology Biomarker Development, Genentech Inc., South San Francisco, CA, USA; ^3^ Research Pathology, Genentech Inc., South San Francisco, CA, USA

**Keywords:** MET, HGF, gastroesophageal cancer, companion diagnostic, immunohistochemistry, gene amplification

## Abstract

Aberrant activation of the HGF/MET signaling axis has been strongly implicated in the malignant transformation and progression of gastroesophageal cancer (GEC). MET receptor overexpression in tumor samples from GEC patients has been consistently correlated with an aggressive metastatic phenotype and poor prognosis. In preclinical GEC models, abrogation of HGF/MET signaling has been shown to induce tumor regression as well as inhibition of metastatic dissemination. Promising clinical results in patient subsets in which MET is overexpressed have spurned several randomized studies of HGF/MET-directed agents, including two pivotal global Phase III trials. Available data highlight the need for predictive biomarkers in order to select patients most likely to benefit from HGF/MET inhibition. In this review, we discuss the current knowledge of mechanisms of MET activation in GEC, the current status of the clinical evaluation of MET-targeted therapies in GEC, characteristics of ongoing randomized GEC trials and the associated efforts to identify and validate biomarkers. We also discuss the considerations and challenges for HGF/MET inhibitor drug development in the GEC setting.

## INTRODUCTION

Gastroesophageal adenocarcinoma (GEC) is a common and highly morbid malignancy accounting for ~1 million cases and in excess of 700,000 cancer-related deaths annually [1]. Peak GEC incidence occurs in the seventh decade and the disease is approximately twice as common in men compared to women. GEC can be sub-divided according to histological appearance into diffuse (undifferentiated) and intestinal (well differentiated) types according to the Lauren classification [2]. There is marked geographic variation with respect to epidemiology and clinical presentation. Approximately 70% of deaths due to GEC occur in developing countries with the highest incidence noted in Asia, Eastern Europe and South America and the lowest rates seen in the United States and Western Europe. While the global incidence of distal gastric adenocarcinoma has steadily declined over time, the incidence of distal oesophageal or gastroesophageal junction (GEJ) tumors have risen sharply in the Western world over the last two decades while remaining relatively uncommon in Asia [3].

Because of its insidious nature, patients newly diagnosed with GEC often present with advanced incurable disease. For those presenting with potentially resectable cancer that undergo surgery with curative intent in conjunction with perioperative chemotherapy or post-operative chemoradiation, overall survival ranges from 30-35% at five years and recurrence within two years following surgery is commonplace [4-6]. Thus, a majority of patients with GEC will ultimately be treated for metastatic disease.

Patients with metastatic GEC (mGEC) can be subdivided into two populations based on HER2 expression status [7]. HER2-positive tumors represent 10-15% of cases and are defined by overexpression of HER2 protein with or without concomitant amplification of its gene ERBB2. For patients with HER2-positive disease, the phase III ToGA trial demonstrated clinically and statistically significant improvements in response rate, progression-free survival (PFS) and OS with the addition of trastuzumab to a cisplatin–fluoropyrimidine doublet (median OS 13.8 versus 11.1 months, HR 0.74, 95% CI, 0.60–0.91; P = 0.0048) [8]. Trastuzumab is now a standard first-line treatment option for patients with HER2-positive advanced or metastatic GEC.

For patients with HER2-negative GEC, doublet or triplet combination chemotherapy remains the mainstay of treatment [7, 9, 10]. For such patients, prognosis remains dismal with median survival following first-line treatment ranging from 9-11 months and a 5-year survival rate of less than 10% [11, 12]. In patients of adequate performance status, second-line chemotherapy has been associated with proven improvements in OS and quality of life compared with best supportive care [7]. In the second-line setting, ramucirumab, a monoclonal antibody targeting VEGFR2 (Lilly) has been recently been shown to extend survival and may become a standard targeted treatment option for patients who have relapsed following first-line treatment.

Effective delivery of conventional cytotoxic therapy is challenging in the setting of GEC by virtue of both substantial disease-related morbidity and the advanced age of patients [13]. These challenges in cytotoxic drug delivery coupled with its limited efficacy means that novel therapeutic modalities are urgently needed to improve clinical outcomes for patients with advanced GEC.

As with other cancers, recent progress in the molecular profiling of GEC has lead to the design of several targeted therapies that are currently in clinical development [13, 14]. Gastric tumours are thought to be molecularly diverse and harbour alterations in several key oncogenes and kinase pathways that may be amenable to pharmacologic inhibition [15, 16]. With the exception of trastuzumab (Herceptin®, Genentech) in HER2-positive GEC and potentially ramucirumab in the relapsed setting, recent Phase III studies of other agents targeting oncogenic mediators such as VEGF-A, EGFR and mTOR in non-enriched patient populations have not been shown to improve survival [13, 17-19]. Of the remaining druggable targets thought to play a role in GEC, agents targeting the MET receptor tyrosine kinase are the currently being subjected to intensive clinical investigation.

This Review will focus on the anti-MET agents currently in late stage clinical development in advanced/metastatic GEC. The Review summarizes the available data as well as the current status of ongoing randomized studies of MET-directed agents. Since identification of tumours most likely to respond to MET pathway blockade is a key component of therapeutic development, we also discuss the issues and challenges associated with identification of MET biomarkers to aid patient selection.

### MET signaling and gastric oncogenesis

MET is a transmembrane receptor tyrosine kinase (RTK) for which hepatocyte growth factor (HGF) is the only known ligand. MET is predominantly expressed on cells of epithelial origin but is also found on non-epithelial tissues such as endothelium, neuronal cells, melanocytes and hematopoietic cells. MET activation induces complex cellular signaling mediated through a variety of transduction pathways driven by a diverse array of adaptors and downstream effectors (for review see Gherardi *et al* [20]). MET primarily signals through RAS-MAPK and PI3K-Akt pathways to evoke pleiotropic cellular processes including motility, survival, proliferation, morphogenesis and angiogenesis that collectively orchestrate a biological program known as “invasive growth” [20-22]. Under physiological conditions, MET-driven invasive growth is tightly regulated and plays a key role in tissue growth and repair. Not surprisingly, cancer cells are able to hijack the invasive growth program in order to propagate an invasive and metastatic phenotype [20]. Aberrant HGF/MET activation occurs in multiple types of malignancies, including GEC, via several mechanisms including overexpression, focal gene amplification, gene copy number gain, activating mutations, RTK transactivation and autocrine or paracrine signaling (www.vai.org/met) [20, 21, 23].

Dysregulated HGF/MET signaling is commonly seen in GEC. Signal activation by HGF in GEC cell lines and tumor models promotes tumorigenesis and metastases. The potentiated capacity for metastatic transformation upon MET activation has been linked with an increased capacity for epithelial-mesenchymal transition (EMT) and inhibiting detachment-mediated apoptosis (anoikis) in GEC models [24]. Perturbation of HGF/MET signaling with anti-HGF antibodies or MET kinase inhibitors attenuates both tumor growth and metastatic dissemination in both GEC cell lines and animal models [24-26]. As HGF and MET mutations are exceedingly rare in GEC [27, 28], activation of MET is thought to be primarily a result of receptor overexpression and/or genomic upregulation (gene copy number gain or amplification). Overexpression of MET protein or transcript as measured by immunohistochemistry (IHC) or RT-PCR respectively is relatively common in GEC tissue. Recent retrospective IHC studies on gastric tumor tissues obtained following tumor resections have reported MET overexpression in 4% - 63% of cases [29-34]. On the other hand, focal *MET* gene amplification appears rare in treatment-naïve gastric tumors with reported incidences of between 0 – 5% [31, 35, 36]. MET receptor overexpression, copy number gain or amplification has been associated with a more aggressive phenotype and diminished survival in multiple retrospective patient series. [29, 31, 35-39] *In vitro*, MET-amplified GEC cells are acutely sensitive to HGF/MET pathway blockade [40, 41].

In addition to oncogenesis and malignant transformation, aberrant MET signaling has been associated with *in vitro* resistance to cytotoxic agents known to be active in GEC [42, 43].

Collectively, these data provide a compelling rationale to clinically evaluate HGF/MET inhibitors in the setting of GEC.

### Clinical experience with MET pathway inhibitors in GEC

**Figure 1:**
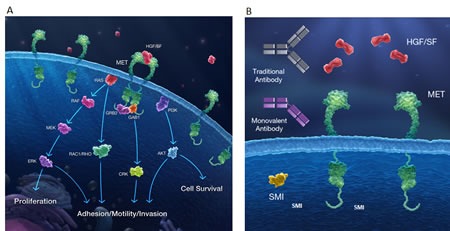
The HGF/MET axis and targeted therapy strategies (A) The MET receptor is activated at the plasma membrane through the binding of HGF to the extracellular domain of MET. Upon dimerization, kinase activation results in trans-autophosphorylation and binding of adaptor proteins, forming scaffolds for recruitment and activation of signaling proteins. MET can then signal through RAS-MAPK, PI3K-AKT, RAC1, and PAK pathways to drive distinct cellular responses including proliferation, survival, motility, invasiveness, and stimulation of angiogenesis. (B) Three pharmacologic approaches are currently being developed as inhibitors of MET signaling including anti-HGF antibodies, monovalent (one-armed) anti-MET antibodies and small molecule MET kinase inhibitors.

Several drugs targeting the HGF/MET signaling axis, including both antibodies and small molecule inhibitors have been evaluated in the clinic. Antibodies directed against either HGF or MET prevent ligand-receptor interaction and consequently impact downstream MET signaling (Figure 1). Small molecule MET kinase inhibitors are generally designed to target the active site of the receptor, inhibiting phosphorylation and recruitment of signaling effectors (Figure 1).

### Monoclonal antibodies

Both rilotumumab (AMG102; Amgen) and onartuzumab (MetMAb; Genentech) are in the latter stages of clinical development for GEC. The primary hypothesis being tested in both studies is whether addition of HGF/MET-targeted therapy to standard platinum-based chemotherapy improves survival in patients with gastroesophageal tumors overexpressing MET.

Rilotumumab is a fully human monoclonal IgG2 antibody that binds HGF and prevents its binding to the MET receptor and subsequent signaling [44]. Onartuzumab is a monovalent (one-armed), humanized monoclonal antibody specific for an epitope in the HGF binding domain of the MET receptor. Onartuzumab prevents HGF binding to MET, inhibiting ligand-induced activation of the intracellular domain, thus blocking downstream MET signaling events [45]. Onartuzumab was specifically designed as a monovalent antibody to avoid agonistic activity that may occur when a bivalent antibody binds two MET molecules [45].

The strongest clinical evidence supporting HGF/MET inhibition in GEC comes from a first-line randomized, placebo-controlled Phase II study of rilotumumab in combination with platinum-based chemotherapy in patients with advanced GEC. In this three arm trial, 121 patients with unresectable locally advanced or metastatic disease were randomized to epirubicin, capecitabine and cisplatin (ECX) plus placebo (n = 39) or ECX plus either 7.5-mg/kg (n=40) or 15-mg/kg rilotumumab (n=42). Patients were primarily drawn from Western countries (Western and Eastern Europe, US and Australia) with only a minority (17%) from Asia. The combination of rilotumumab (low and high doses combined) plus ECX marginally improved both PFS (HR = 0.64) and OS (HR = 0.73) in the intention-to-treat population. Toxicities more frequently seen with rilotumumab included peripheral edema, thrombocytopenia, neutropenia and deep vein thrombosis (DVT). Of note, peripheral edema is a frequently reported toxicity associated anti-HGF/MET antibodies across multiple tumor types and combination regimens [46].

The relationship between MET expression and clinical outcomes were also evaluated in this trial [47]. Archival resection or biopsy specimens suitable for IHC (76% of samples) were tested for MET expression using a proprietary IHC assay. Of the IHC-evaluable patients, 42% were classified as being MET^High^ i.e. weak (1+) or stronger MET staining in >50% of malignant cells. Patients with MET^High^ tumors appeared to experience marked clinical benefit from the addition of HGF-targeted therapy to chemotherapy with respect to PFS and OS compared to those treated with chemotherapy alone (HR_OS_ = 0.29, p = 0.012). On the other hand, worse clinical outcomes were observed in the MET^Low^ patient subpopulation receiving anti-HGF therapy compared to those receiving chemotherapy only (HR = 1.84, *p*-value not reported). Interestingly, a similar trend towards worsened clinical outcomes in MET-negative patients was also observed in study of rilotumumab in patients with castration-resistant prostate cancer [48]. When comparing patients with MET^High^ and MET^Low^ tumors receiving chemotherapy alone, the IHC analysis confirmed that high MET expression is a marker of poor prognosis as suggested by prior retrospective analyses. These data provide clinical proof-of-concept supporting HGF/MET axis targeting in GEC and suggest that MET receptor expression measured by IHC could function as a predictive biomarker for patient selection.

In a Phase I study dose-escalation study of onartuzumab in patients with various locally advanced or metastatic solid tumors, a sustained complete response (>2 years) was noted in a patient with chemorefractory GEC with hepatic metastases [49, 50]. Interestingly, serial correlative blood and tissue studies in this patient showed *MET* gene polysomy, moderate MET expression by IHC and a remarkably high baseline serum HGF level. In addition, intratumoral coexpression of both HGF and MET was observed possibly indicating autocrine signaling [49]. Collectively these laboratory observations may provide important insights into the biologic underpinnings of clinical response to MET blockade in patients with GEC [49].

### Small molecule MET tyrosine kinase inhibitors (TKIs)

Several early phase studies have evaluated anti-MET TKIs in patients with GEC, with mixed results. Crizotinib, a tyrosine kinase inhibitor that targets both ALK and MET tyrosine kinases, was originally developed as a MET-specific inhibitor but has been FDA-approved for use in ALK-positive NSCLC. Single-agent clinical responses to crizotinib were noted in two of four patients with relapsed mGEC and increased MET copy number (≥5 copies) [35]. Conversely, a single-arm Phase II study of foretinib (an oral multikinase inhibitor targeting MET, RON, AXL, TIE-2, and VEGFR2 receptors) failed to demonstrate anti-tumor activity in molecularly-unselected patients with metastatic GEC or those with MET amplified tumors [51]. Similarly, tivantinib (ARQ197, Arqule) monotherapy failed to show clinical activity in a cohort of pre-treated metastatic patients [52]. It should be noted that the mechanisms underlying the antitumor effects of tivantinib are an area of controversy in the literature with recent reports suggesting that antineoplastic activity is primarily attributable to perturbation of microtubule dynamics versus selective MET kinase inhibition [53, 54].

### Ongoing randomized trials of HGF/MET inhibitors in GEC

The evidence linking aberrant HGF/MET signaling to gastric tumorigenesis coupled with encouraging early clinical results has triggered significant clinical development efforts (Table 1). Phase III trials for both rilotumumab and onartuzumab are currently enrolling patients. To our knowledge, no randomized trials involving MET TKIs are currently open at the time of writing.

**Table 1:** Ongoing randomized Phase II and Phase III trials of HGF/MET-targeted drugs in GEC

Study	Patients	Treatment	Stratification	Participating regions	Primary endpoint
NCT01590719 Phase II (YO28252) Sponsor: Roche	1L metastatic GC/GEJ adenocarcinoma (n=123)	mFOLFOX6 ± onartuzumab	Histologic subtype (Lauren) Prior gastrectomy	USA Asia-Pacific (not Japan)	PFS (ITT & MET-positive)
NCT01662869 Phase III (YO28322, MetGastric) Sponsor: Roche	1L metastatic MET-positive GC/GEJ adenocarcinoma (n=800)	mFOLFOX6 ± onartuzumab	MET expression (IHC) Prior gastrectomy Geographic region	USA Western Europe Eastern Europe Asia-Pacific	Overall survival (ITT) Overall survival (MET IHC 2+/3+)
NCT01697072 Phase III (RILOMET-1) Sponsor: Amgen	1L metastatic or unresectable locally advanced MET-positive GC/GEJ adenocarcinoma (n=450)	ECX ± rilotumumab	Extent of disease ECOG PS (0 vs. 1)	USA Western Europe Eastern Europe	Overall survival
NCT01443065 Phase II (MEGA) Sponsor: UNICANCER	1L metastatic or unresectable locally advanced GC/GEJ adenocarcinoma (n=165)	mFOLFOX6 Rilotumumab + mFOLFOX6 Panitumumab + mFOLFOX6	Extent of disease Histologic subtype (signet ring/diffuse vs. intestinal/mixed) Study center.	France only	4-month PFS rate

### Rilotumumab

RILOMET-1 is a randomized, global, double-blind, placebo-controlled Phase III study of rilotumumab in combination with ECX as first-line treatment for advanced MET-positive GEC (NCT01697072) [55]. Patients (planned *n*=450) are randomized 1:1 to ECX (epirubicin 50 mg/m^2^ IV on day 1, cisplatin 60 mg/m^2^ IV on day 1, and oral capecitabine 625 mg/m^2^ twice daily on days 1−21) plus double-blind rilotumumab (15 mg/kg IV) or placebo on a three-weekly cycle. Patients can receive up to ten cycles of ECX, with Rilotumumab or placebo continued until disease progression. Randomization is stratified according to disease extent (locally advanced or metastatic) and ECOG score (0 vs. 1). Eligibility is restricted to patients with MET positive and HER2 negative tumors.

The primary endpoint is OS. Key secondary endpoints include PFS, objective response rate (ORR), OS in MET expression tertiles, safety, and pharmacokinetics. RILOMET-1 is being conducted in approximately 180 sites in Australia, Europe, Africa, North America and South America.

The MEGA (*Met or EGFR inhibition in Gastroesophageal Adenocarcinoma*) study is a randomized Phase II study evaluating the addition of either Rilotumumab or panitumumab (anti-EGFR monoclonal antibody) to mFOLFOX6 as first-line treatment for advanced GEC. Patients from 30 French sites (planned n=165) are randomized 1:1 to mFOLFOX6 alone or combined with either panitumumab (6 mg/kg) or rilotumumab (10 mg/kg). Randomization is stratified by extent of disease (locally advanced vs. metastatic), histologic subtype (signet ring/diffuse vs. intestinal/mixed) and study center. The primary endpoint is 4-month PFS and key secondary endpoints include ORR, PFS, OS and safety.

### Onartuzumab

MetGastric is a randomized placebo-controlled, international Phase III study in patients with previously untreated metastatic GEC (NCT01662869) [56]. Patients (planned *n*=800) are randomized 1:1 to receive either mFOLFOX6 (oxaliplatin: 85 mg/m^2^, IV, day 1; leucovorin: 400 mg/m^2^, IV, day 1; 5-FU: 400 mg/m^2^ bolus followed by 2400 mg/m^2^ over 48 hours starting on day 1) with onartuzumab (10 mg/kg, IV, on day 1, every 14 days) or mFOLFOX6 plus placebo. A maximum of 12 cycles of mFOLFOX6 are permitted. From cycle thirteen onwards, onartuzumab or placebo is continued until disease progression. Randomization is stratified by MET expression status, world region (Asia Pacific vs. Rest of World) and history of prior gastrectomy. Only patients with tumors centrally classified as both HER2-negative and MET-positive (by IHC) are eligible. For the purposes of determining eligibility, a MET positive tumor is defined when ≥50% of malignant cells express MET (cytoplasmic and/or membranous staining) at weak, moderate or strong intensity.

The co-primary endpoint of this study is OS in all patients (ITT) and a subgroup of patients with MET IHC scores of 2+ or 3+. Secondary endpoints include PFS, ORR, safety as well as correlative tissue and biomarker studies.

YO28252 is a multicentre, randomized, placebo-controlled Phase II study of onartuzumab in combination with mFOLFOX6 being run in parallel with MetGastric (NCT01590719). Patients (planned n=120) from the US or Asia-Pacific are randomized 1:1 to mFOLFOX6 in combination with either placebo or onartuzumab. While the Phase II study design and patient population is largely similar to MetGastric, there are some important differences. Unlike MetGastric, patients are not required to have MET-positive disease to be eligible for participation as the primary purpose of the study is to evaluate the clinical profile of onartuzumab in both MET-positive and MET-negative tumors. Randomization is according to Lauren histologic subtype (intestinal/not evaluable vs. diffuse/mixed) and history of prior gastrectomy. The primary endpoint is PFS in both the ITT population and those defined prior to unblinding as MET-positive by IHC. Key secondary endpoints include OS, ORR, safety and pharmacokinetics.

### MET biomarkers in GEC

**Figure 2:**
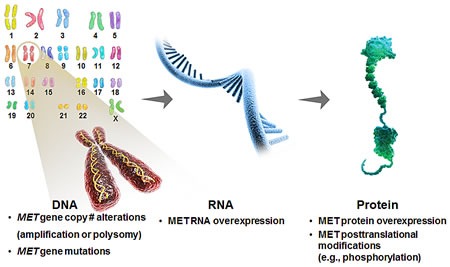
Possible biomarker strategies to identify MET-driven tumors

One of the key challenges associated with the development of targeted therapeutics is identifying tumors that are sensitive as well as patients likely to derive clinical benefit [14]. Critical to this process is the identification and validation of potential predictive biomarkers to tailor treatment. The development of biomarker assays with good specificity and sensitivity to detect these markers in clinical specimens, as well as the inclusion of such tests in both early stage and registration-enabled clinical trials to determine the clinical utility of the diagnostic test is critical. The frequency of MET pathway aberrations and their prognostic potential in GEC support the development and use of pathway-related biomarkers in clinical development (Figure 2). In GEC, multiple biomarker platforms are being evaluated in clinical studies including intra-tumoral HGF/MET expression and alterations in *MET* gene copy number [57].

### MET overexpression

MET protein expression using IHC has been extensively studied in GEC with most studies reporting that MET overexpression is associated with poor patient survival (www.vai.org/met) [38]. Some, but not all, of these studies have suggested a relationship between MET overexpression and histologic subtype, more advanced TNM stage or the incidence of local or distant metastases (www.vai.org/met). In the absence of clinical validation there is currently no consensus on scoring criteria for MET IHC tests. Studies using IHC to evaluate the prognostic impact of MET expression have primarily utilized retrospective analyses of variably sized and annotated clinical cohorts, resulting in a high degree of variability in the published findings with respect to the prevalence of MET overexpression and/or its prognostic implications. Potential sources of variability include the use of different sample types, inter-reader variation, primary and secondary antibodies, staining protocols, scoring methods as well as differences in tissue processing and storage [57, 58]. Furthermore, the IHC reagents used in these studies come with varying degrees of validation with respect to specificity and sensitivity. The source of the diagnostic tissue sample could also be important to consider. Almost all published MET IHC studies have been conducted using tissue derived from gastric tumor resections. Since a large body of patients with GEC present with advanced disease and do not proceed to surgery, it is likely that a significant proportion of diagnostic testing would be conducted on biopsy specimens and not resected samples. It is conceivable that differences may exist in MET expression levels measured in biopsy specimens compared to that seen in resected tissue. Differences in IHC staining between biopsy and resection samples with respect to other IHC markers have been noted [59-61] and if similar observations are made with MET; this could have implications for the use of MET IHC as a companion diagnostic.

**Figure 3:**
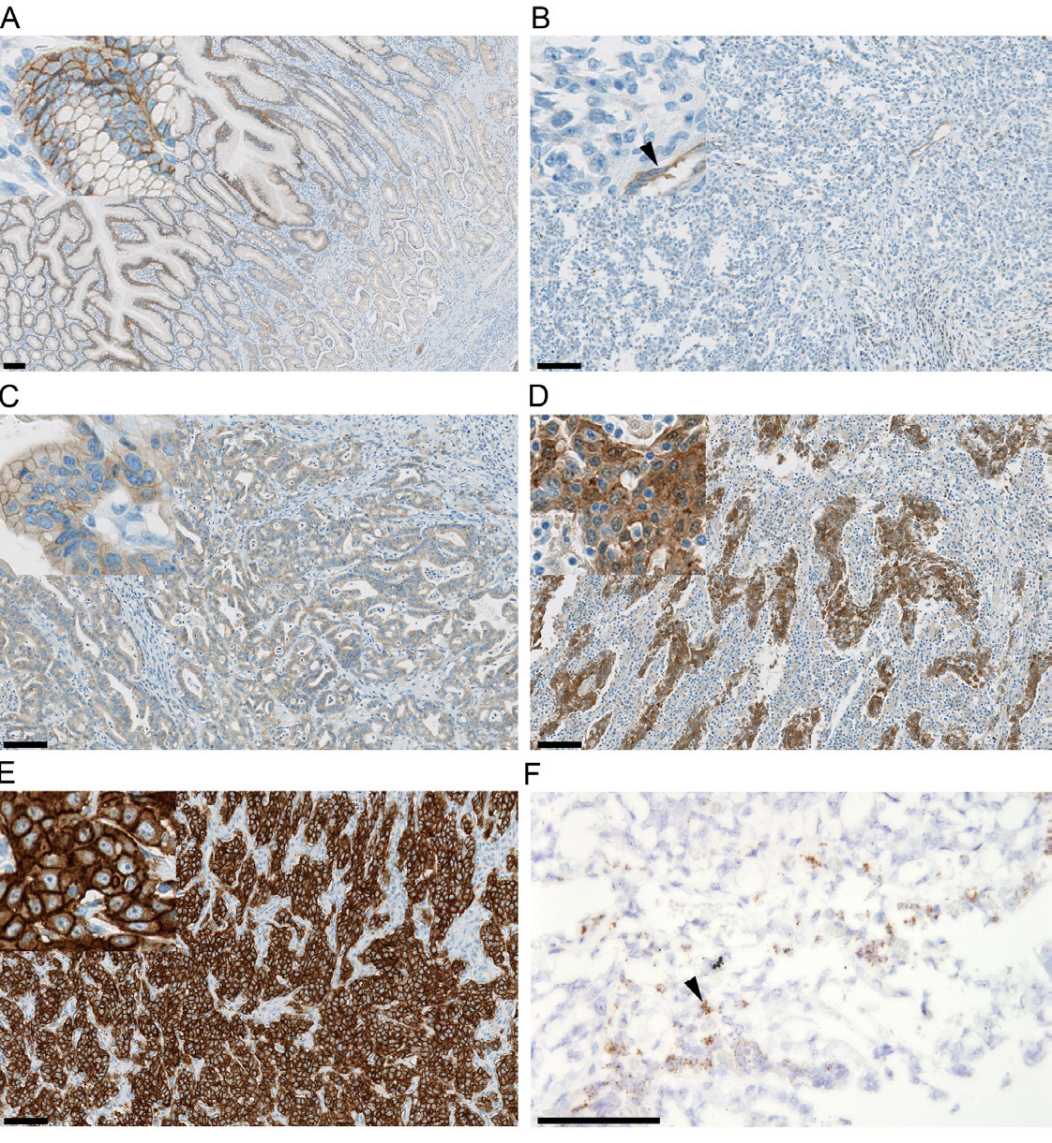
MET and HGF expression in gastric tumor tissue Representative immunohistochemistry exemplifying mild to moderate immunoreactivity in normal foveolar epithelium (A), and gastric cancers with negative (B), mild (C), moderate (D) and strong (E) cytoplasmic and/or membranous MET intensity scores. Vascular immunoreactivity is indicated by the arrowhead (B). Immunoreactivity is shown by brown DAB chromogen deposition against a blue haematoxylin counterstain. Representative in situ hybridization for HGF (F) in a gastric cancer with focal (arrowhead) high expression (3+) in stromal cells. Probe hybridization is shown by the brown chromogen dots against a blue haematoxylin counterstain. Bar = 100 μm, insets for A-E at 5× magnification relative to the main image.

In clinical development, both rilotumumab and onartuzumab are being developed in GEC using a strategy of identifying MET-positive patients by means of a centrally conducted IHC assay on FFPE tissue (Figure 3). For both drugs, the IHC test is being developed as a companion diagnostic. This strategy is based on Phase II studies in NSCLC (onartuzumab) and GEC (rilotumumab) suggesting that MET overexpression is predictive of clinical benefit from anti-HGF/MET therapy [47, 62, 63].

Study OAM4558g was a randomized Phase II study in which patients with relapsed NSCLC were randomized to receive onartuzumab plus erlotinib (Tarceva®, a small-molecule inhibitor of EGFR) or erlotinib alone [62]. In order to evaluate MET expression, an IHC assay was developed utilizing the rabbit monoclonal antibody SP44 (Ventana Medical Systems, Tucson, AZ; cat No. 790-4430) [57]. Due to intratumoral heterogeneity an IHC scoring algorithm was developed incorporating estimates of the proportion of malignant cells showing staining for MET at pre-defined intensity levels. MET IHC was performed on archival resection or biopsy specimens in all randomized patients. MET-positive cases were defined prospectively (prior to study unblinding) as those in which >50% of tumor cells expressed MET at moderate or strong Cytoplasmic and/or membrane intensity. Analysis of efficacy showed that clinical benefit (PFS and OS) was confined to patients with MET-positive tumors [62]. Conversely, clinical outcomes were worse in MET-negative patients treated with onartuzumab plus erlotinib. As a result of this observation the design of the pivotal Phase III study (MetLung; NCT1234567) was restricted to only MET-positive patients [64].

Collectively these data suggest that MET IHC is a valid biomarker with which to select patients for MET-targeted therapy. Despite the encouraging nature of these data sets, limitations must be considered. Namely both Phase II studies had a small sample sizes and did not prospectively stratify based on MET expression status (definition of MET positivity was determined before unblinding but after random assignment). Several outstanding questions remain with respect to the use of MET IHC. Rigorous clinical validation of the diagnostic cut-off point used to define MET positivity will be critical given the detrimental outcomes seen in the Phase II setting in MET-negative patient subsets [47, 48, 62]. Proper selection and validation of cut points for both the extent and intensity of MET staining will be critical. Phase II data so far indicate that using a proportional cut-off of >50% may provide the best discriminative power to select MET-positive patients for treatment with onartuzumab or rilotumumab. For both agents, application of either a more stringent (e.g. 90%) or a less stringent (e.g. 10%) diagnostic cut-off appeared inferior [47, 65]. Differences exist with respect to the intensity of MET IHC staining between different tumor types, which may prove important when validating staining intensity cut-off values. For example, MET staining in GEC cases appears generally weaker and shows a more pronounced cytoplasmic component compared to NSCLC specimens when scored using the SP44 antibody (HK, personal observation) [66]. It is unclear if this variance between cancer types is driven by differences in biology and/or pre-analytical variables such as sample type and tissue processing/fixation [67]. As a consequence of differences seen in the nature and spectrum of MET expression in gastric versus lung tumors, Genentech are employing a broader definition of MET positivity for patient selection in the pivotal onartuzumab trial in GEC (MetGastric) trial compared to MetLung i.e. MET IHC score ≥ 1+ (MetGastric) vs. ≥ 2+ (MetLung).

### MET gene amplification and copy number increase

Aberrations in gene copy number in malignant cells can be driven by either gene amplification or polysomy. Gene amplification refers to a copy number gain for a specific gene (or group of genes) on a given chromosome arm without a change in copy number for genes located in other regions of the chromosome, whereas polysomy gives rise to a copy number gain for a given gene as a result of the presence of extra copies of the entire chromosome [68]. In preclinical models, *MET* gene amplification results in constitutive activation of the MET receptor and an oncogenic addiction to the MET signaling pathway thereby rendering gastric tumor cells acutely sensitive to HGF/MET axis inhibition [40, 41]. While MET amplification is relatively common in GEC cell lines [40], it is has typically been observed in <5% of patient tumor samples [29, 35-37, 69]. Although clinically rare, *MET* amplification has been correlated with an increased frequency of distant metastasis, more extensive TNM stage and diminished survival in retrospective clinical series [29, 35]. *MET* copy number gain has been reported in up to 30% of clinical cases and may be associated with poor prognosis [36, 37]. Preliminary clinical studies of anti-MET therapy in patients with GEC appear to confirm that MET amplification or high-level copy number gain is rare and may not be predictive of clinical benefit [47, 51]. This is supported by a recent analysis by Koeppen et al [65], in which MET copy number (cut-off ≥ 5 copies/cell by FISH) was inferior to MET IHC staining as a predictive marker of benefit from onartuzumab in patients with NSCLC. Retrospective studies in GEC cases suggest that MET copy number maybe positively correlated with protein overexpression measured by IHC suggesting that high gene copy number leads to high protein expression in this subpopulation [29, 31]. Ultimately, the usefulness of MET gene amplification as a predictive biomarker as well as the concordance between amplification and protein expression remain to be established in large-scale randomized trials.

**Table 2:** Comparison of MetGastric and RILOMET-1 Phase III trials. Key differences in study design are highlighted in bold text

Trial design	MetGastric	RILOMET-1
Anti HGF/MET drug	Onartuzumab	Rilotumumab
Patient population	Previously untreated HER2-negative, MET-positive metastatic gastric or GEJ adenocarcinoma	Previously untreated HER2-negative, MET-positive **unresectable locally advanced** or metastatic gastric/GEJ adenocarcinoma
Estimated sample size	~800 patients	~450 patients
Chemotherapy backbone	**mFOLFOX6**	**ECX**
Geographic involvement	Europe, Americas & **Asia-Pacific** (including Australia)	Europe, Americas, South Africa & Australia
Primary endpoint	Co-primary OS (ITT & MET 2+/3+)	OS
Diagnostic partner	Ventana	Dako
IHC antibody	SP44	MET4

### HGF expression

HGF presents the only known ligand for the MET receptor [20]. Intratumoral HGF protein expression measured using IHC or *in situ* hybridization (Figure 3F) as well as systemic levels of HGF has been shown to be elevated in GEC, often associated with poor prognosis [24, 70-72]. It is conceivable that increased local levels of HGF protein may be a relevant indicator of aberrant MET pathway signaling activity not necessarily reflected in systemic HGF levels. In epithelial-derived tumors, HGF expression is primarily restricted to the stromal cell population. However, in rare cases, tumor cells can express HGF, leading to an autocrine loop-type mechanism of activation of MET. In preclinical models, autocrine expression of HGF correlates with active MET signaling and can predict efficacy with MET-targeting agents in the absence of exogenous human HGF [73]. Intratumoral co-expression of both HGF and MET mRNA, possibly indicative of autocrine signaling, was associated with reduced survival and an increased risk of peritoneal dissemination in a cohort of Japanese patients with GEC [24].

Although increased local levels of HGF could lead to the enhanced activation of the MET signaling pathway, these data should be cautiously interpreted. Reagents to evaluate intratumoral levels of HGF protein have not been vigorously validated with respect to sensitivity and specificity and it remains unclear how systemic levels of HGF in the serum/plasma relate to HGF levels and pathway activity in the tumor microenvironment. It remains to be seen if HGF expression with gastric tumors can be reliably measured and if it proves to be a clinically useful biomarker to select patients for anti-MET therapy.

### Outlook and future directions

The HGF/MET signaling axis appears to play an important role in the development and malignant progression of gastroesophageal cancers, particularly in tumor invasiveness and metastasis. Monoclonal antibodies targeting either HGF or the MET receptor are currently in Phase III trials, the results of which will ultimately clarify the role of MET inhibition in the GEC setting. The main challenges facing the effective use of HGF/MET-targeted agents for cancer treatment are optimal patient selection, diagnostic and pharmacodynamic biomarker development, the identification and testing of rational drug combinations and further understanding of the toxicities associated with pathway inhibition.

Defining the patient population most likely to benefit from HGF/MET-targeted therapy is imperative. Multiple biomarker platforms are currently being investigated for this purpose in GEC, the most advanced being IHC. While preliminary data from patient subsets in randomized Phase II trials have shown that high MET protein expression as measured by IHC can differentiate patients who may benefit from MET pathway blockade, the success of MET IHC as a predictive biomarker has not been universal in all tumor types [48]. Susceptibility to pre-analytical variables such as sample type, tissue fixation and processing and the subjectivity of interpretation can be liabilities for IHC. These considerations coupled with the detrimental outcomes seen in patients with low levels of MET expression treated with MET-targeted drugs will demand stringent quality control with respect to the performance and interpretation of IHC in GEC [47, 48, 62]. IHC assays to determine HER2 status in GEC have been in clinical practice as companion diagnostic assays for around three years and have taught us about the benefits and challenges of such an approach. Ongoing clinical studies will also shed light on the usefulness of biomarker platforms other than MET IHC, including HGF expression (IHC, *in situ* hybridization and Real Time PCR), MET amplification and blood-based markers.

Should anti-MET therapy prove to be clinically effective, an understanding of the toxicity implications of MET antagonism will be essential. To date HGF/MET-targeted agents, in particular monoclonal antibodies appear to be relatively well tolerated. Peripheral edema has been associated with treatment with all monoclonal antibodies targeting HGF or MET in combination with various cytotoxic and targeted therapies across multiple tumor types [46]. A recent trial of onartuzumab in triple negative breast carcinoma reported peripheral edema in ~60% of patients randomized to receive onartuzumab [74] The etiology of MET inhibitor-induced edema is unclear, but may be attributable to an attenuation of HGF-mediated signaling in the vascular endothelium. In physiological conditions, HGF in the endothelium helps to protect against VEGF-induced endothelial hyper-permeability. Perturbation of HGF/MET signaling could disrupt this balance resulting in endothelial leak. Interestingly, an increased incidence of venous thromboembolism has also been observed in the setting of HGF/MET inhibition possibly supporting the notion that anti-HGF/MET drugs can disrupt the functioning of the vascular endothelium. Since HGF/MET signaling has been implicated in physiological processes such as tissue growth/repair, hematopoiesis and glucose metabolism; it is possible that large-scale randomized trials of HGF/MET inhibitors may unearth additional toxicity signals such as myelosuppression, mucosal injury, wound healing complications or disturbances in glucose homeostasis [75-79].

The complexity and diversity of HGF/MET signal transduction coupled with the high degree of interplay between MET and other membrane receptors, as well as its role in resistance suggest that combination treatment approaches will be most fruitful.

In the context of GEC, combinability of HGF/MET-targeted agents with chemotherapy will be imperative. Both RILOMET-1 and MetGastric are utilizing different platinum-based cytotoxic combinations (ECX vs. mFOLFOX6) and it remains to be seen if the difference in chemotherapy backbone will impact the outcome of the respective trials. Preclinically, aberrant MET signaling has been implicated in resistance to both cisplatin and oxaliplatin [42, 80]. The clinical question of whether or not the choice of platinum agent in the setting of HGF/MET inhibition is important will be informed by the MEGA and RILOMET-1 studies, which are combining HGF-targeted therapy with mFOLFOX6 and ECX respectively.

A wealth of data indicates a high degree of co-expression and cross-talk with respect to MET and HER family members, which include HER1/EGFR, HER2 and HER3. Amplification or overexpression of HER2 occurs in 10 – 15% of gastroesophageal adenocarcinomas and HER2-targeted therapy (trastuzumab) is approved for use in advanced GEC [7]. While HER2-directed treatment has improved outcomes for eligible patients, additional treatment options are still required in this setting. All ongoing randomized trials of MET-directed treatments are currently excluding those with HER2-positive disease, primarily because of both differences in standard of care and lack of data supporting the combinability of anti-HER2 and anti-MET antibodies. Preclinical data could support such combinations studies. In HER2 amplified GEC cell lines, HGF-mediated MET activation is able to rescue tumor cells from EGFR/HER2 inhibition, an effect that could be abrogated by knockdown or pharmacologic inhibition of MET [81]. While there appears to be minimal overlap between HER2 and MET overexpression in treatment naïve GEC [32], it is possible that compensatory MET upregulation could occur during treatment with anti-HER2 therapy resulting in resistance. This hypothesis could be tested clinically through a trial combining a MET inhibitor with an anti-HER2 agent.

Anti-angiogenic therapy is likely to become a standard-of-care treatment for relapsed mGEC based on results of a Phase III trial of ramucirumab, a VEGFR2-targeted monoclonal antibody [82]. HGF/MET signaling is a potent inducer of endothe-lial cell growth and promotes angiogenesis and lym-phangiogenesis *in vitro* and *in vivo* and is though to be a key regulator of the angiogenic switch [83]. HGF/MET and VEGF– VEGFR2 cooperate in inducing angiogenesis *in vitro* and *in vivo* through activation of common signaling intermediates. Collectively, these data could support combination trials of HGF/MET-directed therapy with anti-angiogenic agents. The results of ongoing trials of MET-targeted drugs with anti-VEGF therapy in other tumor types will shed light on the usefulness of this approach [46, 84].

Should HGF- or MET-directed therapy prove to be an effective treatment option for patients with GEC, experience with other RTK inhibitors suggests that resistance will invariably develop even in the subset of cancers that initially derive clinical benefit [85]. *In vitro* mechanisms of resistance to MET-targeted agents include mutation in the MET activation loop, compensatory upregulation of HER kinase signaling as well as amplification of *MET* and *KRAS* [85-87]. In the event that MET inhibitors enter the clinic, rationally designed combination trials will be needed in order to abrogate resistance to anti-HGF/MET therapy and further improve clinical outcomes.

It is presently unclear to what extent targeting MET or HGF might impact the efficacy of HGF/MET axis inhibition. The results of RILOMET-1 and MetGastric will provide some clinical insight into this issue should the results of the respective trials be divergent. Of interest, a randomized trial of ficlatuzumab (a humanized antibody against HGF; Aveo) in combination with an EGFR inhibitor in NSCLC failed to show efficacy in patients with high MET expression Mok et al [88]. However, subgroup analysis suggested that clinical benefit was enhanced in patients with either stromal HGF expression or low MET expression. These results are in contrast with other Phase II studies in which high tumor MET expression was predictive of clinical benefit from HGF/MET-directed antibodies and might suggest that receptor versus ligand targeting could be important depending on the tumor type and/or combination partner.

Although GEC is a global disease, it is not uniform. There are marked differences in patient demographics, treatment practices and treatment outcomes in GEC patients in different countries and regions [89]. It is not clear if such geographic heterogeneity extends to biological differences. In the setting of anti-angiogenic therapy, patients from Asia (primarily Japan and Korea) represented 49% of the total patient population enrolled in the AVAGAST trial and seemed to gain less from the addition of bevacizumab than patients in the rest of the world [17]. Conversely, in the REGARD study, Asian patients represented only 8% of the study population [82]. Collectively, the outcome of these trials and the apparent regional differences in outcome evoke multiple questions about global disease heterogeneity and its importance in targeted therapy drug development. It remains to be seen if such geographic heterogeneity will extend to outcomes with respect to HGF/MET-directed therapy. Of note, while RILOMET-1 is not involving countries from the Asia-Pacific region, MetGastric is randomizing both Asian and non-Asian patients. Should the outcomes of these respective pivotal trials be different, this could indicate clinically relevant geographic heterogeneity with respect to HGF/MET biology.

## CONCLUSION

Targeting the HGF/Met axis has significant clinical potential in GEC, which will hopefully be realized in the context of well-conducted clinical trials, the development and clinical validation of pathway biomarkers and rational mechanism-based treatment combinations.
